# Coping Strategies, Psychological Impact, and Support Preferences of Men With Rheumatoid Arthritis: A Multicenter Survey

**DOI:** 10.1002/acr.23422

**Published:** 2018-04-16

**Authors:** Caroline A. Flurey, Sarah Hewlett, Karen Rodham, Alan White, Robert Noddings, John R. Kirwan

**Affiliations:** ^1^ University of the West of England Bristol UK; ^2^ Staffordshire University Stoke‐on‐Trent UK; ^3^ Leeds Beckett University Leeds UK; ^4^ University Hospitals Bristol NHS Trust Bristol UK; ^5^ University of Bristol Bristol UK

## Abstract

**Objective:**

To investigate the existence and distribution of 2 typologies (termed “factors”) of men with rheumatoid arthritis (RA) identified through our previous Q‐methodology study (n = 30) in a larger sample of men with RA, and whether differences in psychosocial impact or support preferences exist between the 2 factors, and between men and women with RA.

**Methods:**

A postal survey was sent to 620 men with RA from 6 rheumatology units across England, and the support preferences section of the survey was given to 232 women with RA.

**Results:**

A total of 295 male patients (47.6%) and 103 female patients (44.4%) responded; 15 male participants had missing data, and thus 280 were included in the analysis. Of these, 61 (22%) were assigned to factor A (“accept and adapt”), 120 (35%) were assigned to factor B (“struggling to match up”), and 99 (35%) were unassigned. The two factors differed significantly, with factor B reporting more severe disease, less effective coping strategies, and poorer psychological status. For support, men favored a question and answer session with a consultant (54%) or specialist nurse (50%), a website for information (69%), a talk by researchers (54%), or a symptom management session (54%). Overall, women reported more interest in support sessions than men, with ≥50% of women reporting interest in nearly every option provided.

**Conclusion:**

Some men accept and adapt to their RA, but others (43%) report severe disease, less effective coping, and poor psychological status. Men's preferences for support are practical, with a focus on expanding their knowledge.

## Introduction

Rheumatoid arthritis (RA) is a long‐term condition, characterized by painful, swollen, and stiff joints and fatigue 1, 2. RA affects more women than men (approximately 30% of all patients are men) 3 and may take a different course in women compared to men, with male sex being a potential predictor of remission in RA 4. A recent literature review 5 found that the majority of gender‐based research on the psychosocial impact and self‐management in rheumatology either addressed the differences between men and women, reflecting the preponderance of women with the condition, or focused solely on women. Very little research has focused solely on men, and there is no consensus on whether gender affects a person's ability to cope with RA. Qualitative research has begun to address the experiences and coping styles of men with RA and its impact on their masculine identity 6, 7, 8, 9, suggesting a need to renegotiate masculine identity and rewrite scripts on masculinity 10 to adapt to life with RA.

**Significance & Innovations**

- There are at least 2 groups of men with rheumatoid arthritis (RA), one (≥43%) of which appears to be struggling to accept and cope with RA and is not being served by current self‐management interventions because of the personal coping strategies used by this group.
- Men's preferences for support are practical, with a focus on expanding their knowledge about their condition and how to manage it.
- Women report interest in self‐management options more than men, indicating a need to be wary of men being outnumbered in mixed‐gender support interventions.

In a previous phase of this research, a Q‐methodology study used qualitative and quantitative methods to group men with RA (n = 30) according to their level of agreement with statements about living with and managing their condition 11. Two groups (termed “factors” in Q‐methodology) were identified; the first group (Q‐factor A: accept and adapt) were able to take control of other areas of their lives to enable them to accept the loss of control due to RA, and found ways to adapt to their condition. The second group (Q‐factor B: struggling to match up) tried to continue taking part in the masculine activities they had taken part in before their diagnosis, despite the further pain this caused them. However, they were reluctant to accept physical or emotional support from others.

Evidence from several long‐term conditions indicate that there are gender differences in the impact of illness and in the ways of coping with it 12, 13, suggesting that men need a health strategy tailored to them 14. The similarities between the qualitative and Q‐methodology findings in men with RA, and those identified as specific to men with other long‐term conditions, suggest that men with RA may need their own tailored support, which has not previously been investigated. Due to their different approach to coping, the men in Q‐factors A and B may require different support approaches from each other and from women with RA. However, it is possible that there may be overlap in the support preferences of women and the men in Q‐factor A, who seem able to accept and adapt to their condition.

The nature of qualitative and Q‐methodology research involves relatively small numbers of participants; it would therefore be useful to understand whether the issues raised by the qualitative work, and the 2 groups of men identified in the Q‐methodology study, exist in a wider population of men with RA. If these 2 groups do exist, it is important to understand whether they have different preferences for support provision, and whether these preferences differ sufficiently from those of female patients to justify the development of a support intervention tailored toward men with RA. Thus, in this study we had 2 aims: to investigate whether factors A and B (from the Q‐methodology study) are generalizable across men with RA, and whether these factors can be explained by demographics, disease status, coping strategies, or psychological status; and to understand whether there is a difference in the support preferences of the men in factor A, men in factor B, and women with RA.

## Subjects and methods

A questionnaire using validated measures and items created by the research team was developed based on themes and issues that emerged as important in the previous qualitative and Q‐methodology studies. Two questionnaires were mailed to male participants in a single survey packet. The first collected demographic and clinical information, and asked patients to use a numeric rating scale (NRS) to respond to the statements used in the Q‐methodology study that distinguished the 2 Q‐methodology factors from each other. Distinguishing statements were chosen if the average scores between the 2 factors were sufficient to highlight the differences in the experience of living with RA 15. Statements were included if there was a ≥4‐point difference between the composite scores for each factor, as this was a natural cutoff point at which there were a manageable number of statements for participants to rate. From the original sample of 64 statements, 12 statements were included in the survey, with each factor represented by 6 distinguishing statements.

The first questionnaire also measured coping strategies, acceptance of illness, perceived stress, depression and anxiety, and mental well‐being. The measures used for each assessment are shown in Table 1. The second questionnaire concerned patient preferences for self‐management support, including mode of delivery and practical issues (e.g., time of day). Options for self‐management support came from previous qualitative work 8, 9 and a systematic review of effectiveness and acceptability of self‐management support for men with long‐term conditions 16, 17. To assess whether men and women have different preferences for support, the questionnaire for female participants contained the sections on demographic and clinical information, and self‐management support preferences. The Q‐methodology distinguishing statements were not included since they were developed with men, and may not be appropriate for a female population. The (longer) male questionnaire was piloted with a male patient research partner (RN), who gave advice about the order of some items and indicated that the questionnaire took 30–40 minutes to complete.

**Table 1:** Items included in the questionnaire^a^

Section	Validated	Measure	Description
About you	No	Age	Open response
	No	Comorbidities	Open response
	No	Marital status	Tick options
	No	Employment status	Tick options
	No	Level of physical activity in job	NRS 1–5
	No	Level of autonomy in job	NRS 1–5
About your RA	No	Medication	Open response
	No	Disease duration	Open response
	Yes	Patient‐based disease activity score 40	Patient‐reported measure of disease activity
Your experience of RA	No	Distinguishing statements from 2 Q‐methodology factors	21 statements taken from our previous research 20; NRS 0–10 asking how much each statement relates to their experience of living with RA
Coping with RA	Yes	Medical Coping Modes Questionnaire 41, 42	19 items measuring the extent to which participants are using 3 coping strategies (confrontation, avoidance, and acceptance‐resignation) in dealing with their RA
Your feelings about your RA	Yes	Acceptance of Illness Scale 43	8 items measuring the extent to which participants have accepted their RA
Life in general	Yes	Short Form Perceived Stress Scale 44	4‐item measure of stress that focuses on elements of control
	Yes	Hospital Anxiety and Depression Scale 45	14‐items measuring levels of anxiety and depression
	Yes	Short Form Warwick and Edinburgh Mental Well‐Being Scale 46, 47	7‐items measuring mental well‐being

a NRS = numeric rating scale; RA = rheumatoid arthritis.

To capture a range of views, participants were recruited through rheumatology units in 6 regional hospitals across England, selected to reflect diverse geographical locations and serving different communities in relation to urbanity/rurality and socioeconomic status. Patients were included who were over 18 years old and with a confirmed diagnosis of RA from their rheumatologist reported in their records. To recruit sufficient male participants within a reasonable time, at each hospital a member of the local team screened their database for male RA patients. A questionnaire was then mailed to either a random selection of 100 patients (using a random number generator in Excel) or every male RA patient in the database (whichever was smaller). For the female participants, at each hospital a member of the local team handed questionnaires to consecutive female patients attending an outpatient appointment. A convenience sample of female participants is sufficient to broadly compare the support preferences of men and women. If there is a clear difference between support preferences, this would be apparent in any group of women approached, without the need for strategic sampling. Both male and female participants were assigned a study number, and if no response was received, they were sent a reminder approximately 2 weeks later. All responses were returned directly to the central research team in a prepaid envelope.

Questionnaire data were entered into SPSS for Windows, and Brown's factor index scoring method 15, 18 was used to investigate the likely membership of each survey study participant to the 2 Q‐methodology factors. In Q‐methodology, data analysis uses correlation and by‐person factor analysis, meaning that statistical analysis is not performed by variable, but by person. People correlate with others with similar opinions based on their Q‐sorts. Q‐methodology therefore results in the grouping of expressed opinion profiles based on the similarities and differences in which the statements are arranged by each participant 19. Thus, to retain this by‐person analysis, Brown's factor index scoring method was chosen to identify how common the previously identified experiences (Q‐factors A and B) are in the wider population, and whether they relate to patients’ coping styles, psychological status, and support preferences.

Participant scores on the numeric rating scale (NRS) for each distinguishing statement are used to calculate a standardized index score for each participant for each factor, which indicates to what extent the participant is associated with each factor. The scoring procedure for 1 participant (M049) is shown in Table 2. The Q‐factors, the selected distinguishing statements, and the Q‐factor scores of the statements in the original Q‐methodology study are shown in the first 4 columns. In column 5, the mean item score for each statement, representing mean agreement with the statements across participants, is shown. For example, “keeping active helps me manage my RA symptoms” has a mean score of 7.1, indicating that male patients in the overall sample tend to agree with this statement. NRS scores of statements with negative factor scores were reverse scored. Statement index scores and factor index scores were calculated for each participant. The statement index score is calculated as the product of the absolute value of the factor score (which is fixed across participants, as it originates from the previous Q‐methodology study) and the item score (which varies between participants, based on their NRS scores). For example, the statement “I still socialize as much as I used to before having RA” had a ranking of +3 for Q‐factor A in the Q‐methodology study, and participant M049 gave this statement an NRS score of 4, giving participant M049 a statement index score of 12. Thus, the statement index score takes into account the weighting given to each item within the relevant Q‐factor as determined by the previous Q‐methodology study 11. Factor index scores were calculated for each factor as the sum of the statement index scores of each participant for the relevant factor. Participant M049 had a factor index score of 100 for factor A and 333 for factor B, indicating that while this participant had some agreement with factor A, there was clearly stronger agreement with factor B. The mean statement and factor index scores for the participants in the current study are shown in Table 3.

**Table 2:** Calculation of Brown's factor index score using participant M049 as an example^a^

Distinguishing statements by factor	Factor A	Factor B	Mean NRS statement, all	M049 NRS statement	M049 statement index	M049 factor index
Factor A						100
Keeping active helps me manage my RA symptoms	+5	+1	7.1	8	40	
I am able to find different ways of doing things I want to, or different activities to replace those I have lost	+5	−1	6.8	6	30	
I still socialize as much as I used to before having RA	+3	−3	6.7	4	12	
RA has taken away my independence	−5	+2	6.7^b^	2^b^	10	
I worry more about money now that I have RA	−5	+1	6.9^b^	0^b^	0	
Since being diagnosed with RA I have lost a lot of confidence	−4	0	6.5^b^	2^b^	8	
Factor B						333
I feel frustrated because of my RA	−2	+7	5.3	10	70	
I get angry because of my RA	−7	+6	4.2	9	60	
I sometimes feel guilty about the effect my RA has on the people around me	−1	+5	4.6	10	50	
My faith helps me cope with my RA	−1	−7	7.7^b^	9^b^	63	
If I need a tool/device/gadget to help with my RA, I will make it myself	0	−5	7.1^b^	9^b^	45	
I don't mind having to ask a stranger for help when I need to	+1	−5	6.8^b^	9^b^	45	

a NRS = numeric rating scale; RA = rheumatoid arthritis.

b Item scores of statements with a negative factor score were reverse scored.

**Table 3:** Mean statement and factor index scores for Q‐methodology factor A and factor B*

Distinguishing statements by factor	Factor A	Factor B	Mean statement score†	Statement index score, mean ± SD	Range	Factor index score, mean ± SD	Factor index score, range
Factor A						203.1 ± 64.4	24–310
Keeping active helps me manage my RA symptoms	+5	+1	7.1	35.4 ± 12.1	0–50		
I am able to find different ways of doing things I want to, or different activities to replace those I have lost	+5	−1	6.8	33.8 ± 12.9	0–50		
I still socialize as much as I used to before having RA	+3	−3	6.7	19.8 ± 18.8	0–60		
RA has taken away my independence	−5	+2	6.7	33.3 ± 16.4	0–60		
I worry more about money now that I have RA	−5	+1	6.9	34.5 ± 16.6	0–50		
Since being diagnosed with RA I have lost a lot of confidence	−4	0	6.5	26.1 ± 12.8	0–40		
Factor B						209.4 ± 66.5	32–350
I feel frustrated because of my RA	−2	+7	5.3	37.2 ± 23.1	0–70		
I get angry because of my RA	−7	+6	4.2	25.2 ± 20.8	0–60		
I sometimes feel guilty about the effect my RA has on the people around me	−1	+5	4.6	23.2 ± 17.7	0–50		
My faith helps me cope with my RA	−1	−7	7.7	54.2 ± 22.3	0–70		
If I need a tool/device/gadget to help with my RA, I will make it myself	0	−5	7.1	35.3 ± 17.5	0–50		
I don't mind having to ask a stranger for help when I need to	+1	−5	6.8	33.9 ± 16.8	0–50		

*RA = rheumatoid arthritis.

†Item scores of statements with a negative factor score were reverse scored.

*T*‐tests, Mann‐Whitney tests (as appropriate) and chi‐square tests were used to assess demographic, clinical, and psychosocial differences between factors A and B. Distributions of responses were used to describe support preferences. Chi‐square tests were used to test whether there were any differences in support preferences between factors A and B, and between male and female participants.

## Results

### Are factors A and B generalizable across men with RA, and can they be explained by demographics, disease status, coping strategies, or psychological status?

Responses were received from 295 of 620 male patients (47.6%) and 103 of 232 female patients (44.4%). A total of 280 male participants fully completed the Q‐methodology NRS and were therefore included in the analysis. Of these, 61 (22%) had factor index scores that indicated that their opinions belong to factor A (“accept and adapt”), 120 (43%) could be assigned to factor B (“struggling to match up”), and 99 (35%) had less than 1 standard deviation between their factor index scores and were therefore unassigned to a factor. The proportions of factor A, factor B, and unassigned male participants in the present survey study were similar to those of the original Q‐methodology study.

Demographic and clinical data are shown in Table 4. For male participants, these data and coping strategies and psychological status are presented combined and separately for the groupings according to Q‐methodology factors. There were no significant differences between the men assigned to factor A and those assigned to factor B in age, comorbidities, marital status, or disease duration. However, participants assigned to factor B (“struggling to match up”) were less likely to be retired (*P* < 0.000) and if working were significantly less likely to consider their role to be particularly physically active (*P* = 0.040) or autonomous (*P* = 0.007). Those assigned to factor B reported a significantly higher patient global score (*P* < 0.001), and more of them were receiving biologic therapies (*P* = 0.010).

**Table 4:** Demographic, clinical, and psychosocial data by gender and Q‐methodology factor^a^

Variable	Men, factor A (n = 61)	Men, factor B (n = 120)	Men, unassigned (n = 99)	Men, total (n = 280)	Women, total (n = 103)
Age, years					
Mean ± SD	68 ± 10.1	64 ± 10.9	67 ± 11.0	65.7 ± 10.9	62 ± 12.0
Range	37–85	28–82	32–90	28–90	28–83
Comorbidities, %					
Yes	61	71	68	68	66
Marital status, %					
Married	69	77	77	75	65
Single	10	8	6	7	3
Divorced	6	4	6	7	6
Widowed	8	5	7	5	15
Living with partner	7	4	4	5	10
Prefer not to say	0	2	0	1	1
Employment status, %					
Full time	21	26	24	24	11
Part time	13	7	13	10	18
Retired	66	54^b^	62	60	55
Unemployed (due to RA)	0	12	1	5	13
Unemployed (other)	0	0	0	0	2
Prefer not to say	0	1	0	1	1
Level of PA in job, %					
None	3	1	3	2	5
A little	5	3	8	5	8
Some	2	6	10	6	3
Quite a bit	10	15	12	13	7
A great deal	16^c^	10	4	9	5
No answer	64	64	63	65	72
Level of autonomy in job, %					
None	2	1	3	2	1
A little	0	6	1	3	3
Some	2	8	7	6	5
Quite a bit	10	14	15	13	13
A great deal	22^d^	7	11	11	6
No answer	64	64	63	65	72
Disease duration, years					
Mean ± SD	15.0 ± 10.1	14.6 ± 11.1	14.2 ± 12.2	14.6 ± 11.2	12 ± 11.2
Range	1–37	1–53	0.5–69	0.5–69	0.2–55
PtGA, mean ± SD	18.3 ± 17.3	51.0 ± 24.9^b^	35.2 ± 26.3	38.2 ± 27.1	47.4 ± 26.8
PDAS, mean ± SD	3.3 ± 0.6	4.7 ± 0.9	4.0 ± 1.1	4.2 ± 1.9	4.5 ± 1.0
Medication, %					
DMARDs	50	92	79	81	88
Biologics	18	40^e^	29	31	40
Steroids	26	28	31	30	31
None	8	4	5	5	5
Coping strategies, mean ± SD					
Confrontation	16.0 ± 3.2	17.3 ± 3.2^f^	17.1 ± 3.7	17.0 ± 3.5	–
Avoidance	13.5 ± 3.3	15.7 ± 3.0^b^	14.5 ± 3.5	14.8 ± 3.4	–
Resignation	7.3 ± 1.0	8.8 ± 1.7^b^	7.7 ± 1.5	8.1 ± 1.6	–
Acceptance	35.0 ± 5.2^b^	21.6 ± 6.7	28.9 ± 7.0	27.1 ± 8.4	–
Depression, %					
Case	2	22^b^	5	11	–
Borderline case	2	25^b^	8	15	–
Noncase	96	53^b^	87	74	–
Anxiety, %					
Case	2	22^b^	10	13	–
Borderline case	2	23a	14	15	–
Noncase	96	55a	76	72	–
Perceived stress, mean ± SD	2.5 ± 2.7	6.6 ± 3.3^b^	4.3 ± 3.1	4.9 ± 3.5	–
Mental well‐being, mean ± SD	27.7 ± 4.9^b^	21.7 ± 4.2	25.1 ± 5.1	24.3 ± 5.3	–

a Factor A defined as “accept and adapt” and factor B defined as “struggling to match up.” RA = rheumatoid arthritis; PA = physical activity; PtGA = patient global assessment; PDAS = patient‐based disease activity score; DMARDs = disease‐modifying antirheumatic drugs.

b *P* < 0.001.

c *P =* 0.040.

d *P* = 0.007.

e *P* = 0.010.

f *P* = 0.15.

Participants assigned to Factor B were more likely to use the coping strategies of confrontation (*P* = 0.15), avoidance (*P* < 0.001), and resignation (*P* < 0.001) and were less accepting of their RA (*P* < 0.001). Participants assigned to factor B reported poorer psychological status, with significantly more cases or borderline cases of both anxiety and depression than those assigned to factor A (*P* < 0.001 for both), as well as higher levels of perceived stress (*P* < 0.001) and lower levels of mental well‐being (*P* < 0.001).

### Is there a difference in the support preferences of men in factor A, men in factor B, and women with RA?

Preferences for self‐management support are shown in Table 5. The most popular methods of support selected by men with RA (reaching ≥50%) were: a one‐on‐one session with a consultant (83%), specialist nurse (80%), or physical therapist (53%); a question‐and‐answer session with a consultant (54%) or specialist nurse (50%); a website for information (69%); an organized talk by research experts (54%); or an education session on symptom management (54%). Factor B participants were significantly more likely than factor A participants to select an education session on managing stress and anger (factor A 18%, factor B 34%) or an education session on symptom management (factor A 54%, factor B 63%). Men were least interested in a one‐on‐one (28%) session or a question‐and‐answer session with another patient (20%). Although these were not the most popular options with the female participants (49% and 45%, respectively), female participants were significantly more interested in interacting with another patient than male participants were (*P* = 0.003 and *P* < 0.001, respectively).

**Table 5:** Preferences for self‐management support services compared by gender and Q‐methodology factor^a^

	Men, factor A (n = 61)	Men, factor B (n = 120)	Men, unassigned (n = 99)	Men, total (n = 280)	Women, total (n = 103)
Mode of delivery for support^b^					
Discussion group					
About experiences of RA	32	35	30	34	59^c^
To exchange tips about RA	36	44	40	41	64 (10th)^c^
To discuss research (e.g., papers)	34	24	31	29	53^c^
One‐on‐one consultation					
With consultant	82 (1st)	82 (1st)	84 (1st)	82 (1st)	86 (1st)
With specialist nurse	79 (2nd)	80 (2nd)	81 (2nd)	79 (2nd)	87 (2nd)
With physical therapist	55 (5th)	53 (6th)	51 (6th)	53 (6th)	56
With occupational therapist	40	49 (10th)	40	43	59^d^
With psychologist	27	21	26	23	39^e^
With another patient	32	26	27	27	49^c^
Question‐and‐answer session					
With consultant	55 (6th)	54 (5th)	53 (5th)	54 (5th)	67 (7th)
With specialist nurse	50 (9th)	51 (7th)	48 (7th)	51 (7th)	67 (7th)^d^
With physical therapist	41	31	37	35	43
With occupational therapist	34	31	29	31	45
With psychologist	30^e^	20	20	22	45^c^
Organized talks					
Lifestyle experts	54 (7th)	45	42 (8th)	45 (8th)	76 (4th)^c^
Expert patients	30	24	28	27	55^c^
Research experts	63 (4th)^f^	49 (9th)	56 (4th)	55 (4th)	70 (6th)^f^
Education sessions					
Managing stress/anger	18	34^g^	23	27	48^c^
Managing symptoms	54 (8th)	63 (4th)^h^	42 (8th)	53 (6th)	75 (5th)^c^
Physical activity sessions					
To develop skills (e.g., balance)	36	35	40	37	64 (10th)^c^
To improve fitness	39	51 (8th)	39	44 (9th)	53
Organized game (e.g., walking football)	23	25	23	24	24
Raising awareness of RA event					
Attend	39	38	34	36	65 (9th)^c^
Take part in	18	22	18	19	27
Help organize	13	12	17	14	25
Online services					
To read information	73 (3rd)	65 (3rd)	71 (3rd)	69 (3rd)	81 (3rd)
To read other patients’ stories	46 (10th)	45	42 (8th)	44 (9th)	64 (10th)^i^
To read questions and answers	21	20	30	24	27
To communicate with other patients about emotions	20	23	24	23	44^c^
To communicate with other patients about practical issues	27	27	30	28	49^c^
Chat room	18	17	20	18	27
Message board	49	39	39	42	59^j^
Time of day for support services					
Early morning (pre–9 am)	12	6	13	10	6
Morning (9 am–midday)	44	32	45	40	30
Lunchtime (midday–2 pm)	18	26	28	24	33
Afternoon (2–5 pm)	28	32	32	31	43
Evening (after 5 pm)	24	29	24	25	22
Frequency					
Single on/off group	17	9	20	15	10
Fixed time period (e.g., 1/week for 6 weeks)	6	17	17	14	17
No fixed commitment, an advertised timetable to dip into	77	76	64	72	76
Gender of the group					
Same gender	6	4	9	7	4
Mixed, equal number of men and women	14	18	31	19	17
Mixed, my gender should outnumber the other	2	0	0	1	2
Mixed, do not mind if the other outnumbers mine	22	11	9	13	8
No preference	56	67	61	62	68
Other people					
A service for people with RA only	22	13	20	17	9
Would like to invite a friend/family member	37	50	32	41	54
Would not bring someone, but would not mind a group open to friends/family	41	38	48	42	38
Motivators					
An appointment letter	57	47	54	52	61
Invitation from rheumatologist	63	71	67	68	69
Invitation from specialist nurse	57	61	51	57	71
Reimbursement of travel costs	22	25	19	21	32
Money or vouchers for attendance	7	12	14	11	18
Location away from the hospital	9	21	20	17	30

a Values are percentages unless otherwise indicated. RA = rheumatoid arthritis.

b Rankings of the 10 most popular modes of delivery in each group are shown in parentheses.

c Comparison between men and women, *P* < 0.001.

d Comparison between men and women, *P* = 0.006.

e Comparison between men and women, *P* = 0.003.

f Comparison between men and women, *P* = 0.010.

g Comparison between factors, *P* = 0.046.

h Comparison between factors, *P* = 0.010.

i Comparison between men and women, *P* = 0.001.

j Comparison between men and women, *P* = 0.007.

Generally, women reported being interested in support sessions more than men, with ≥50% of women reporting interest in nearly every support option provided. Thus, there were no options selected more highly by men than women. The preferred time of day for a support intervention for men was in the morning (9 am to midday, 39%) and for women it was in the afternoon (2–5 pm, 43%). Both men and women would prefer a modular approach to self‐management support, with an advertised program that they could access at their convenience (72% and 76%, respectively). The majority of both men (63%) and women (68%) reported no preference over group gender. Further, only 17% of men and 9% of women reported that a support group should be for people with RA only. Conversely, 41% of men and 55% of women would like to have the option of inviting a friend or family member, while 42% of men and 35% of women report that although they would not bring someone they would not mind if others did. Men and women reported being more likely to be motivated to attend a self‐management session if they were sent an appointment letter (men 52%, women 61%), or invited to attend by their rheumatologist (men 68%, women 69%) or specialist nurse (men 56%, women 71%).

## Discussion

The current study found that in a large and diverse sample of patients with RA there are 2 types of coping styles among men. One group (factor B: “struggling to match up”) reported using less effective coping strategies, having less acceptance, and lower psychological well‐being than the other group (factor A: “accept and adapt”). The experience of RA and coping styles of men in factor A support the suggestion that men perceive ill health as a threat to their masculine identity, and addressing health concerns can challenge their health‐related beliefs of men being self‐reliant and resilient 20. However, dealing with health concerns can be perceived as taking action to gain control when men's health status begins to threaten their independence 21, which may be the cognitive mechanism being employed by the participants in factor A.

The characteristics of participants in factor B indicate that these men would be less likely to engage with health care and therefore less likely to take part in a research study. It is therefore possible that we may have underrecruited participants who would be factor B participants (52.4% of invited participants declined to take part). Therefore, the size of the factor B group as reported here (43% of participants) may be an underestimate of the number of men with RA who have these coping strategies.

These groups had previously been identified as factors in a Q‐methodology study 11, but they might have been a reflection of the relatively small sample size of that study. The current results show that these groups do exist in a wider sample of men with RA, and that a significant proportion of male RA patients (43% in the current study) are in need of an appropriately targeted support or self‐management intervention from their rheumatology team. This adds to the more general perception that men need their own health strategy 14. The preferences for support among men belonging to both factors A and B and those participants who were unassigned to either factor are broadly similar, indicating that a common method of support provision across male patients may be acceptable. Recent research in other conditions suggests that support services need to be sensitive to gender considerations to ensure that interventions do not undermine masculine values, and address men's concerns 13.

The most popular form of support identified by men was a one‐on‐one session with their rheumatologist, specialist nurse, or physical therapist. This reflects the current provision of care, but men were also interested in a question‐and‐answer session with their rheumatologist or specialist nurse, opportunities to hear about current research, and education sessions for symptom management. This is similar to evidence from studies of other long‐term conditions suggesting that support services for men should have a practical focus 16 and provide opportunities to gather new information 22, 23 and that men use information exchange as a form of emotional support 24. Despite this finding, men are underrepresented in trials focusing on symptom management in RA (e.g., in a fatigue management program, 85.4% of the participants were female) 25. Further, despite those in factor B reporting poor psychological well‐being, only 34% of these patients recognized the need for an education session on managing stress and anger. It may therefore be necessary to take a gender‐sensitized approach to the advertising and delivery of an intervention for men with RA to increase engagement; an approach of this kind has been successfully carried out in an intervention for obesity (Football Fans in Training) 26.

Male participants were less interested than women in hearing from other RA patients, which may reflect men's preferences for seeking out information rather than experiences 16. Although this may call into question the appropriateness of providing interventions involving co‐delivery with patients for men, previous qualitative work found that co‐facilitation by a patient research partner in focus groups was helpful for engaging men in discussion 8. Female participants indicated greater acceptance than males of all support options, which is in line with previous findings in RA 27. Engaging in health practices for well‐being rather than for physical health can be perceived as less masculine 28. Thus, the men in this study may show less interest in support than women as they are engaging in the masculine ideal of being “strong and silent” 29.

Previous research comparing interactions of men and women in online forums about breast cancer (aimed at women) and prostate cancer (aimed at men) found that quantitatively women dominated both forums. Qualitatively, while the men made attempts to accommodate their communication to the norms of the opposite gender, the women did not 30. Thus, despite both male and female participants reporting no clear preference for a single‐gender group, it may be important to provide men with RA with an all‐male intervention to enable them to engage according to masculine norms. Evidence from the obesity literature suggests that male‐only groups are qualitatively different from mixed‐gender groups, with different levels of engagement, styles of language, and success 31, 32.

Both male and female participants reported a preference for a modular approach to support, whereby different topics would be covered in each session and patients could access support according to an advertised timetable. This approach may be more complex to evaluate in a randomized controlled trial, but is a potential way forward for intervention delivery.

Further, both male and female participants reported being more likely to attend a self‐management intervention if they received an appointment letter or personal recommendation from their rheumatologist or specialist nurse. This supports the view that self‐management should be seen as integral to treatment, rather than as a “nice optional extra” 33. This study asked participants about support preferences, but it is not known how this would translate into uptake in clinical practice. It is possible that some of the responses given reflect social desirability, such as male participants reporting no gender preference for the group. However, the responses were anonymous, and participants were advised of this. It is possible that those men who may have belonged to factor B were underrecruited, as the very characteristics included in factor B may have reduced the proportion of participants taking part who could be assigned to this factor. This potential recruitment bias would reduce the likelihood of identifying factor B, which nevertheless emerged. Thus, the size of factor B as reported here (43% of participants) may be an underestimate of the number of men with RA who use these coping strategies. This survey sampled patients across 6 hospitals in England, thereby accessing a range of disease experiences and care pathways, and it also involved a patient research partner (RN) from design to interpretation. Although this study was conducted in the UK, the literature suggests commonalities in the psychological impact of inflammatory arthritides, such as RA, across Europe and North America 34, 35. Masculinity is thought to be socially constructed 36; thus these results may be specific to Western culture and may not be relevant in a different sociocultural context. However, qualitative studies suggest commonalities between the UK and other European countries on the impact of inflammatory arthritis on masculinity 37, 38, 39. Thus, men's coping strategies and preferences for psychological support may be relevant to patients internationally.

In conclusion, our findings suggest that there are at least 2 groups of men with RA, one of which (≥43% of total) appears to be struggling to accept and cope with their RA and are not being served by current self‐management interventions because of their personal coping strategies. Men's preferences for support are practical, with a focus on expanding their knowledge about their condition and how to manage it. Men reported being more likely to take part in a self‐management session if it were legitimized by their clinical team. Further research should pilot potential self‐management support for men to test appropriate content, delivery style, and recruitment techniques.

## Author contributions

All authors were involved in drafting the article or revising it critically for important intellectual content, and all authors approved the final version to be submitted for publication. Dr. Flurey had full access to all of the data in the study and takes responsibility for the integrity of the data and the accuracy of the data analysis.

### Study conception and design

Flurey, Hewlett, Rodham, White, Noddings, Kirwan.

### Acquisition of data

Flurey.

### Analysis and interpretation of data

Flurey, Hewlett, Rodham, White, Noddings, Kirwan.
